# Lessons Learned from CNV Analysis of Major Birth Defects

**DOI:** 10.3390/ijms21218247

**Published:** 2020-11-03

**Authors:** Alina Christine Hilger, Gabriel Clemens Dworschak, Heiko Martin Reutter

**Affiliations:** 1 Department of Pediatrics, Children’s Hospital Medical Center, University Hospital Bonn, 53127 Bonn, Germany; 2 Institute of Human Genetics, University Hospital Bonn, 53127 Bonn, Germany; 3 Institute for Anatomy and Cell Biology, University Hospital Bonn, University of Bonn, 53115 Bonn, Germany; 4 Department of Neonatology and Pediatric Intensive Care, Children’s Hospital Medical Center, University Hospital Bonn, 53127 Bonn, Germany

**Keywords:** copy number variation, CNV, birth defect, de novo, embryonic development

## Abstract

The treatment of major birth defects are key concerns for child health. Hitherto, for the majority of birth defects, the underlying cause remains unknown, likely to be heterogeneous. The implicated mortality and/or reduced fecundity in major birth defects suggest a significant fraction of mutational de novo events among the affected individuals. With the advent of systematic array-based molecular karyotyping, larger cohorts of affected individuals have been screened over the past decade. This review discusses the identification of disease-causing copy-number variations (CNVs) among individuals with different congenital malformations. It highlights the differences in findings depending on the respective congenital malformation. It looks at the differences in findings of CNV analysis in non-isolated complex congenital malformations, associated with central nervous system malformations or intellectual disabilities, compared to isolated single organ-system malformations. We propose that the more complex an organ system is, and the more genes involved during embryonic development, the more likely it is that mutational de novo events, comprising CNVs, will confer to the expression of birth defects of this organ system.

## 1. Introduction

The prevention and treatment of major birth defects are key concerns for child health. According to “EUROCAT”, the European network of population-based registries for the epidemiological surveillance of congenital anomalies, congenital anomalies are structural defects (congenital malformations, deformations, disruptions and dysplasias) and chromosomal abnormalities. They are the major cause of infant mortality, childhood morbidity and long-term disability. They are also the major cause of embryonic and fetal death. Congenital anomalies are among the leading causes of years of potential life lost and carry a high burden to affected individuals, their families and the community in terms of quality of life, participation in the community and need for services. In Germany, about 25% of all pediatric deaths and about one third of all pediatric hospital admissions are associated with birth defects [1]. Hitherto, for the majority of defects, the underlying cause remains unknown, likely to be heterogeneous [2].

### 1.1. Clinical Presentation

**Figure 1:**
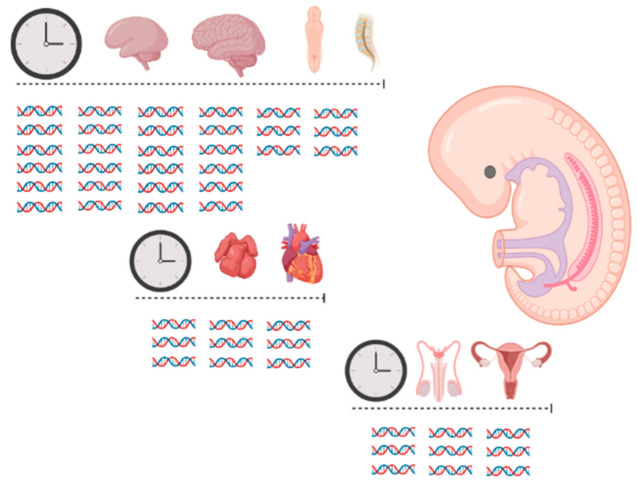
Schematic overview of time frame and involvement of genes for different organ systems during human embryonic development comparing CNS, heart and genital organ development (created with Biorender.com).

According to Rasmussen et al. (2003) [3], the classification of birth defects, respectively a newborn presenting with birth defects, involves the question whether the newborn has the defect of interest as an isolated defect, as one of multiple congenital anomalies (non-isolated) or as a component of a syndrome (non-isolated syndromic). Application of this classification system is necessary to assess if the birth defect is part of a “recognizable pattern” of multiple malformations that is known or presumed to have a specific cause (e.g., a single-gene condition, chromosome abnormality, multifactorial or teratogenic exposure) (Figure 1) [4]. This classification system is independent of the health-related impact of the respective birth defect but is necessary when systematic genetic studies on cohorts of affected individuals are performed.

### 1.2. Causes for Congenital Birth Defects

According to the “Malformation Monitoring Centre Saxony-Anhalt (http://www.angeborene-fehlbildungen.com/)”, it has been estimated that the underlying cause for congenital anomalies or malformations is monogenic in about 10% of cases, due to chromosomal anomalies in about 5% of cases, due to maternal disease in another 5% of cases and probably multifactorial in 20% of cases. In about 60% of cases, the underlying cause remains elusive at the time of writing. The implicated mortality and/or reduced fecundity in many major birth defects suggest a significant fraction of rare recessive or mutational de novo events among the affected individuals. The experience in medical genetics suggests that these mutational de novo events will comprise genomic alterations of different size ranging from small changes affecting single nucleotides to large alterations resulting in losses or gains of several thousand to millions of base pairs.

## 2. Copy-Number Variations (CNVs) and Their Impact on the Expression of Birth Defects

Copy-number variations (CNVs) represent areas of DNA gains or losses. These regions are larger than 1000 bp and represent variable copy numbers in comparison with the reference genome [5]. Prior to the advent of array-based molecular karyotyping, reports describing disease-causing CNVs in individuals with birth defects are rare [6]. While many CNVs contribute only to our genetic variability without any implications for disease expression, the experience in medical genetics suggests that larger de novo CNVs are more likely to be disease associated [7]. Furthermore, it has been suggested that phenotypes with multiple congenital anomalies are more likely to be caused by CNVs than isolated defects [8]. Furthermore, it seems that certain phenotypes are associated with certain CNVs in the genome, independent whether this CNV represents a gain or loss of genetic material, e.g., 22q11.2 deletion or duplication syndrome and congenital heart defects [9,10].

## 3. Experience of Array-Based Molecular Karyotyping (CNV Analysis) in Larger Cohorts of Individuals with Major Birth Defects

### 3.1. CNV Analysis in Individuals with the Bladder Exstrophy Epispadias Complex (BEEC)

In 2010, our group and the group of Agneta Nordenskjöld independently identified three de novo and one inherited 22q11.2 duplication in four unrelated individuals with classic bladder exstrophy (CBE) [11,12]. We then carried out multiplex ligation dependent probe amplification analysis in 217 unreported CBE individuals and identified four additional 22q11.21 duplications. Physical alignment of these duplications revealed a 414-kb phenocritical region harboring 12 RefSeq genes [13]. We then carried out array-based analysis in two further tranches on 110 individuals in 2012 and 169 individuals in 2013. The latter tranche comprised 17 individuals with isolated epispadias only, 126 CBE individuals and 26 individuals with exstrophy of the cloaca, the most severe phenotypic expression of the bladder exstrophy epispadias complex (BEEC). In the first tranche, we detected a de novo duplication (0.9 Mb) involving chromosomal region 19p13.12 [14] (see Table 1). Within the duplication reside several RefSeq genes that are expressed in the cloacal membrane of mouse embryos at E10.5 corresponding to early human urogenital development. In the second tranche, we found eight rare inherited CNVs not present in 1307 in-house controls (frequency < 0.0008) suggesting that some of these CNVs might contribute to the BEEC in a multifactorial disease-model [15]. Overall, we performed high resolution array-based molecular karyotyping in 295 BEEC individuals. While our understanding of the contribution of the detected CNVs to BEEC is currently scant, all CNVs comprise one or more genes that are expressed in urogenital structures of mouse embryos during early development.

### 3.2. CNV Analysis in Individuals with Anorectal Malformations (ARM)

Previously, our group systematically employed array-based molecular karyotyping in a total of 224 isolated (*n* = 5) and non-isolated (*n* = 219) individuals with anorectal malformations (ARM) [16,17,18,19,20,21,22,23]. In individuals with non-isolated ARM, the ARM occurred in most individuals as part of their VATER/VACTERL association. Our analysis detected 13 individuals with a single de novo CNV, likely to be disease causing. Furthermore, we detected six CNVs in independent individuals that were not present in the respective parent available for testing. Because only one parent was available for testing, it remains unknown if the respective CNV occurred de novo or not. Among the de novo CNVs, two deletions comprised chromosomal region 13q31.2 [17]. Furthermore, we detected four CNVs comprising chromosomal region 22q11.2. Here, one deletion and one duplication of chromosomal region 22q11.21 occurred de novo [18,22]. In two further deletions of chromosomal region 22q11.2, only one parent was available for de novo testing [18,23]. Independent CNV studies implicated chromosomal region 22q11.21 with ARM [24,25]. While the reported CNVs do not define a phenocritical region for ARM within chromosomal region 22q11.21, it seems evident that CNVs within this region pose a risk for the expression of human ARM phenotypes (see Table 2).

### 3.3. CNV Analysis in Fetuses with Congenital Anomalies of the Kidney and the Urinary Tract (CAKUT)

Verbitsky et al. reported CNV analysis in 2824 CAKUT cases and 21,498 controls using array-based molecular karyotyping. They identified 45 CNVs at 37 independent genomic loci, known to be causal in CAKUT. In addition, novel CNVs were identified in ~2% of cases, indicating substantial genetic heterogeneity. Sixty-five percent of all identified CNVs in known loci were accounted for by only six loci (1q21, 4p16.1-p16.3, 16p11.2, 16p13.11, 17q12 and 22q11.2). Deletions at 4p16.1-p16.3, 17q12 and 22q11.2 were specific for kidney anomalies [26].

### 3.4. CNV Analysis in Fetuses with Central Nervous System Malformations (CNS Malformations)

#### 3.4.1. CNV Analysis in Fetuses with “Non-Isolated” Brain Malformations

Previously our group systematically employed array-based molecular karyotyping in a total of 33 fetuses with non-isolated brain malformations. In 11 of these fetuses, we identified 15 CNVs, comprising four duplications and eleven deletions. Seven of these 15 CNVs occurred de novo, rendering them likely to be disease-causing. All larger CNVs (>5 Mb) had already been detected by prenatal conventional karyotyping. None of the 15 CNVs was present in 1307 healthy in-house controls (frequency < 0.0008). Among these CNVs, we prioritized six chromosomal regions (1q25.1, 5q35.1, 6q25.3-qter, 11p14.3, 15q11.2-q13.1 and 18q21.1) due to their previous association with human brain malformations or owing to the presence of a single gene expressed in human brain. Prioritized genes within these regions were *UBTD2*, *SKA1*, *SVIP* and *GPR52* [27] (see Table 3).

#### 3.4.2. CNV Analysis in Fetuses with “Isolated” Brain Malformations

Accordingly, our group previously employed array-based molecular karyotyping in a total of 35 fetuses with isolated brain malformations. Here, we detected five disease-causing CNVs in four fetuses involving chromosomal regions 6p25.1-6p25.3, 6q27, 16p12.3, Xp22.2-Xp22.32 and Xp22.32-Xp22.33. Furthermore, we detected a probably disease-causing CNV involving chromosomal region 3p26.3 in one fetus; in addition, in another nine fetuses, we detected 12 CNVs of unknown clinical significance. All CNVs except for two were absent in 1307 healthy in-house controls (frequency < 0.0008). Our data suggest the involvement of the genes *CNTN6* and *KLHL15* in the etiology of agenesis of the corpus callosum, the involvement of *RASD1* and *PTPRD* in Dandy–Walker malformation and the involvement of *ERMARD* in ventriculomegaly [28] (see Table 3).

## 4. “Sense and Sensibility” for the Diagnostic and Scientific Application of Array-Based Molecular Karyotyping in Individuals with Birth Defects

Depending on the phenotype investigated, we observed very different results in our systematic CNV analysis. Although the largest cohort analyzed by systematic CNV analysis was the BEEC (*n* = 295), our analysis identified only two different de novo and likely disease-causing CNVs, respectively. One of the two CNVs, a de novo duplication (0.9 Mb) involving chromosomal region 19p13.12 [14], was found in a single individual only. Interestingly, in seven individuals with CBE, we identified dup22q11.21 as the disease causing CNV. We did not identify a comparable homogeneous finding in any of the other investigated cohorts. This finding might be explained by the fact that the BEEC occurs: (i) mostly isolated with no other organ system involved; and (ii) the phenotypic spectrum is reduced to three phenotypes with CBE being by far the most common phenotype (over 85%) [29].

The second largest cohort that we systematically investigated for disease-causing CNVs consisted of individuals with ARM. Phenotypes within the cohort were highly heterogeneous comprising a small group of individuals with isolated ARM and a large group with non-isolated ARM. Within this large group of non-isolated ARM individuals, the majority presented with normal neurocognitive development and without congenital anomalies of the CNS; some individuals also had impaired neurocognitive development and/or congenital anomalies of the CNS. Within this group, we identified 13 disease-causing CNVs with some of them previously reported in association with both ARM phenotypes and congenital anomalies of the CNS, e.g., 6q1 monosomy [30] or 22q11.2 deletion syndrome [25,31,32,33,34]. This finding might be explained by the fact that ARM have been previously associated as part of the phenotypic spectrum in over 500 entries in the OMIM^®^ (Online Mendelian Inheritance in Man^®^) database (October 2020; 555 entries for anal atresia: https://www.omim.org/) suggesting: (i) ARM to be a common phenotypic feature of many genetic syndromes; and (ii) ARM are far more heterogeneous in origin than for example the BEEC. Furthermore, the phenotypic expression of ARM is by far more complex and diverse [35] compared to the BEEC [36] which could be due to the interplay of more genes involved in embryonic hindgut development compared to embryonic bladder formation and/or whether hindgut development and differentiation requires a longer period of time compared to bladder formation with more gene–gene interactions. The smallest cohort that we systematically investigated for disease-causing CNVs comprised isolated and non-isolated CNS malformations. Here, we investigated in total 68 fetuses with isolated (*n* = 35) and non-isolated (*n* = 33) brain malformations and identified a disease-causing CNV in 22% of cases. This high yield of identified disease-causing CNVs might be due to the fact that at least a third of the human exome is expressed in the human CNS, influencing its development and function [NIH Publication No. 10-5475;2010]. This is the highest proportion of genes expressed in any part of the human body. Furthermore, development of the central nervous system and maturation endures during the complete embryonic period and continues during fetal development. This long developmental period and the large number of genes involved in CNS development make the CNS more vulnerable than any other organ system for de novo mutations of different size throughout the genome (Figure 1).

## 5. The Dilemma of Causality

### 5.1. Genotype-Phenotype Correlation Exemplary for the Chromosome 22q Locus

Microdeletions and duplications of chromosomal region 22q11.2 represent the most common disease-causing CNV in human, leading to malformations and dysfunctions of multiple organs, including heart, CNS and kidney [37]. Multiple segmental duplications on chromosome 22q result in a predisposition to genomic rearrangements [38]. However, the phenotype is highly variable and patients with the same aberration may represent a spectrum rather than a reproducible phenotype, representing interfamilial and intrafamilial variability [39]. Since the correlation between DiGeorge syndrome [MIM: #188400] and microdeletion of chromosome 22q in 1981 [40], more than three decades have passed until the responsible genes or phenocritical regions have been defined. Although the typical 3-Mb 22q11.2 locus harbors more than 90 genes, knowledge about the role of every respective gene is sparse and research has focused on the candidate genes, such as *TBX1*. Haploinsufficiency of the *TBX1* gene is responsible for most of the physical malformations, particularly the heart defects [41]. Later, the *CRKL* gene was identified as the main genetic driver of congenital urinary anomalies [42]. These examples show the importance of animal models for the field of medical genetics. Unraveling the molecular mechanisms has direct implication for clinical practice. This has been demonstrated with the finding of five individuals with variants in *CRKL* with congenital urinary anomalies, putting the *CRKL* gene on the diagnostic list for individuals with isolated birth defect (i.e., congenital urinary anomalies) [42].

### 5.2. Genotype-Phenotype Correlation in CNVs Not Comprising Coding Region

While haploinsufficiency of certain genes in microdeletions and continuous gene deletion syndromes can be assessed by showing the same phenotype genotype correlation of point mutations and deletion (e.g., *CRKL*, or van der Woude [MIM #119300] or Feingold Syndrome [MIM #164280]), this correlation is missing in CNVs affecting non-coding regions. However, disruption of chromatin features such as topologically associated domains (TAD) boundaries can lead to so-called enhancer hijacking, exposing enhancers to new target transcription start sites [43]. TAD Boundary disruption and consecutive misexpression of genes leading to birth defects have been described in limb defects where deletions, inversions or duplications of the *WNT6/IHH/EPHA4/PAX3* locus were shown to be associated with distinct forms of limb malformations [44]. This example shows that the pathogenetic effect of CNVs (and of structural variations) can be far more complex than gene-dosage theories alone.

## 6. The Importance of Functional Studies in Cells and Animal Models

The advent of novel molecular tools and their application in animal models have allowed exciting insights into the complexity of the genome. The CRISPR/Cas9 technology has been applied to investigate the consequence of CNVs and structural variations (e.g., inversions) [45]. These techniques are even more important when CNVs of non-coding regions have to be evaluated. For example, it has been found that duplications of upstream *IHH* regulatory elements were associated with syndactyly and craniosynostosis [46]. Only by the thorough work-up in a mouse model it was possible to assess consequences of deletion or duplication of these regulatory elements [47]. Interestingly, loss of enhancer sequences resulted in growth defects of the skull and long bones. In contrast, gain of enhancer sequences resulted in a dose-dependent upregulation and misexpression of *Ihh*, leading to abnormal phalanges, craniosynostosis and syndactyly.

In a study of branchiooculofacial syndrome [MIM #113620], Laugsch et al. investigated the consequences of an inversion using patient-specific human induced pluripotent stem cells (iPSCs). They demonstrated that the inversion disconnects one *TFAP2A* allele from its cognate enhancers, leading to monoallelic and haploinsufficient *TFAP2A* expression in the patient-specific iPSC-derived neural crest, therefore illustrating the power of iPSC as a model to unveil long-range pathomechanisms in structural variations [48].

## 7. Conclusions

We suggest that the diagnostic yield of CNV analysis among individuals with congenital malformations depends highly on the respective congenital malformation. CNV analysis in isolated congenital malformations, affecting one organ system only, will most likely not identify disease-causing CNVs. CNV analysis in non-isolated complex congenital malformations, comprising CNS malformations or intellectual disabilities, will more likely identify disease-causing CNVs in up to 20% or more of affected individuals. However, the latter depends on the affected organ system. If the affected organ system is complex in nature, it usually requires the involvement of many genes and a larger time frame during embryonic development. Such organ systems are more vulnerable for damaging de novo events than others, simply due the larger number of genes involved for organ development. Examples for such organ systems are the heart and the CNS. Here, the experience in medical genetics shows us that systematic molecular karyotyping will identify disease-causing CNVs, even in a large proportion of individuals with isolated defects [49,50].

## Figures and Tables

**Table 1:** CNV Analysis in Individuals with Bladder Exstrophy Epispadias Complex (BEEC). Abbreviations: duplication (dup), deletion (del).

Phenotype	Study	Individuals	Disease-Causing CNVs
**BEEC**	Draaken et al., 2010, 2013, 2014	N = 295 (295 isolated)	dup19p13.12 7 × dup22q11.21
	**Total Number**	**N = 420**	**Disease-causing CNVs *n* = 8**
			**Cases solved 2%**

**Table 2:** CNV Analysis in Individuals with Anorectal Malformations (ARM). Abbreviations: duplication (dup), deletion (del).

Phenotype	Study	Individuals	Disease-Causing CNVs
**ARM**	Schramm et al., 2011a, Schramm et al., 2011b	*n* = 16 (5 isolated/11 non-isolated)	dup18p11.21–18q12
**ARM and CNS**	Dworschak et al., 2015	*n* = 32 (32 non-isolated)	del6q14.3q16.3, del14q32.2, del17q12q21.2, 2 × del22q11.21
**ARM in the context of the VATER/VACTERL Association**	Schramm et al., 2011b, Hilger et al., 2013, Dworschak et al., 2013, Zhang et al., 2017a	*n* = 176 (176 non-isolated)	dup1q41, dup2q37.3, dup8q24.3, del13q31.2-qter, del17q12, del22q11.21, dup22q11.21
	**Total Number**	***n* = 224**	**Disease-causing CNVs *n* = 13**
			**Cases solved 6%**

**Table 3:** CNV Analysis in Individuals with Central Nervous System Malformations. Abbreviations: duplication (dup), deletion (del).

Phenotype	Study	Individuals	Disease-Causing CNVs
**Central Nervous System Malformations**	Krutzke et al., 2015, Schumann et al., 2016	*n* = 68 (35 isolated)	del1q25.1, del3p26.3, dup5q35.1, del6p25.1-6p25.3, del6q25.3-qter, del6q27, dup9p23, dup11p14.3, del15q11.2-q13.1, del16p12.2, dup17p11.2-17p12, dup18q21.1, delXp22.2-Xp22.32
	**Total Number**	***n* = 420**	**Disease-causing CNVs *n* = 15**
			**Cases solved 22%**
